# Deciphering the Molecular Mechanism of Spontaneous Senescence in Primary Epithelial Ovarian Cancer Cells

**DOI:** 10.3390/cancers12020296

**Published:** 2020-01-27

**Authors:** Martyna Pakuła, Ewa Mały, Paweł Uruski, Anna Witucka, Małgorzata Bogucka, Natalia Jaroszewska, Nicoletta Makowska, Arkadiusz Niklas, Rafał Moszyński, Stefan Sajdak, Andrzej Tykarski, Justyna Mikuła-Pietrasik, Krzysztof Książek

**Affiliations:** 1 Department of Hypertensiology, Angiology and Internal Medicine, Poznan University of Medical Sciences, Długa 1/2 Str., 61-848 Poznan, Poland; mpakula@ump.edu.pl (M.P.); puruski@ump.edu.pl (P.U.); aniaizbicka@gmail.com (A.W.); aniklas@mp.pl (A.N.); tykarski@o2.pl (A.T.); jmikula@ump.edu.pl (J.M.-P.); 2 Poznan University of Medical Sciences Core Facility, Rokietnicka 8 Str., 60-806 Poznan, Poland; emaly@ump.edu.pl (E.M.); mbogucka@ump.edu.pl (M.B.); njaroszewska@ump.edu.pl (N.J.); nicola.makowska@ump.edu.pl (N.M.); 3 Division of Gynecological Surgery, Poznan University of Medical Sciences, Polna 33 Str., 60-535 Poznan, Poland; rafalmoszynski@gmail.com (R.M.); ssajdak@ump.edu.pl (S.S.)

**Keywords:** aging biomarkers, cancer biology, cellular senescence, epithelial ovarian cancer, oxidative stress

## Abstract

Spontaneous senescence of cancer cells remains a puzzling and poorly understood phenomenon. Here we comprehensively characterize this process in primary epithelial ovarian cancer cells (pEOCs). Analysis of tumors from ovarian cancer patients showed an abundance of senescent cells in vivo. Further, serially passaged pEOCs become senescent after a few divisions. These senescent cultures display trace proliferation, high expression of senescence biomarkers (SA-β-Gal, γ-H2A.X), growth-arrest in the G_1_ phase, increased level of cyclins D1, D2, decreased cyclin B1, up-regulated p16, p21, and p53 proteins, eroded telomeres, reduced activity of telomerase, predominantly non-telomeric DNA damage, activated AKT, AP-1, and ERK1/2 signaling, diminished JNK, NF-κB, and STAT3 pathways, increased formation of reactive oxygen species, unchanged activity of antioxidants, increased oxidative damage to DNA and proteins, and dysfunctional mitochondria. Moreover, pEOC senescence is inducible by normal peritoneal mesothelium, fibroblasts, and malignant ascites via the paracrine activity of GRO-1, HGF, and TGF-β1. Collectively, pEOCs undergo spontaneous senescence in a mosaic, telomere-dependent and telomere-independent manner, plausibly in an oxidative stress-dependent mechanism. The process may also be activated by extracellular stimuli. The biological and clinical significance of pEOC senescence remains to be explored.

## 1. Introduction

Cellular senescence refers to a phenomenon in which cells are permanently growth-arrested due to the accumulation of extensive and unrecoverable damage to telomeric and/or non-telomeric DNA. The development of senescence is associated with characteristic changes in gene expression, cell phenotype, and function [1]. Classically, senescence was described and characterized in normal somatic cells; however, recent research provides evidence that it may also occur in cancer cells. Cancerous cells, which initially avoid senescence during immortalization, contain clearly intact pro-senescence mechanisms, which may be re-initiated in response to therapy. The development of a senescence phenotype has been identified in tumors subjected to clinically relevant doses of radiation [2] and chemotherapy, including cisplatin, carboplatin, doxorubicin, and etoposide [3].

At the same time, very little is known about the spontaneous senescence of cancer cells as a therapy independent process. In breast cancer patients who had not received chemotherapy, 10% of tumors displayed senescence-associated β-galactosidase (SA-β-Gal) [4]. The presence of senescent cancer cells was also found in cultured primary breast, colon, and prostate cancer cells, and established breast cancer cells, vulvar cancer, lung cancer, and melanoma cells [5]. Conversely to therapy-induced senescence, the spontaneous senescence of cancer cells is often treated as an artifact and the mechanisms and potential clinical significance of this poorly characterized phenomenon remains unclear. It must be stressed that the senescence of normal cells has been considered a pro-cancerogenic phenomenon. Particularly, senescent cells create an immunosuppressive tissue microenvironment [6] and promote various aspects of cancer cell progression both in vitro and in vivo [7]. Taking this into account, the senescence of cancer cells should be considered from two sides: as a cancer-restricting mechanism, due to the inability of senescent cancer cells to further divide, and as potentially pro-cancerous event, which can reinforce proliferating cancer cells in an autocrine manner. The significance of this process for a given patient remains unknown.

Here, we present a comprehensive analysis of the spontaneous senescence of primary epithelial ovarian cancer (pEOC), a malignancy in which this phenomenon has not been addressed phenomenologically or mechanistically. The study contains results of in vivo observations based on the identification of senescent cancer cells in tumors from patients with pEOC. These findings are followed by in vitro experiments on primary ovarian cancer cells, in which all critical molecular and biochemical parameters characterizing cell growth and senescence are evaluated.

## 2. Results

### 2.1. The Presence and Characteristics of Spontaneously Senescent pEOCs In Vivo and In Vitro

**Figure 1:**
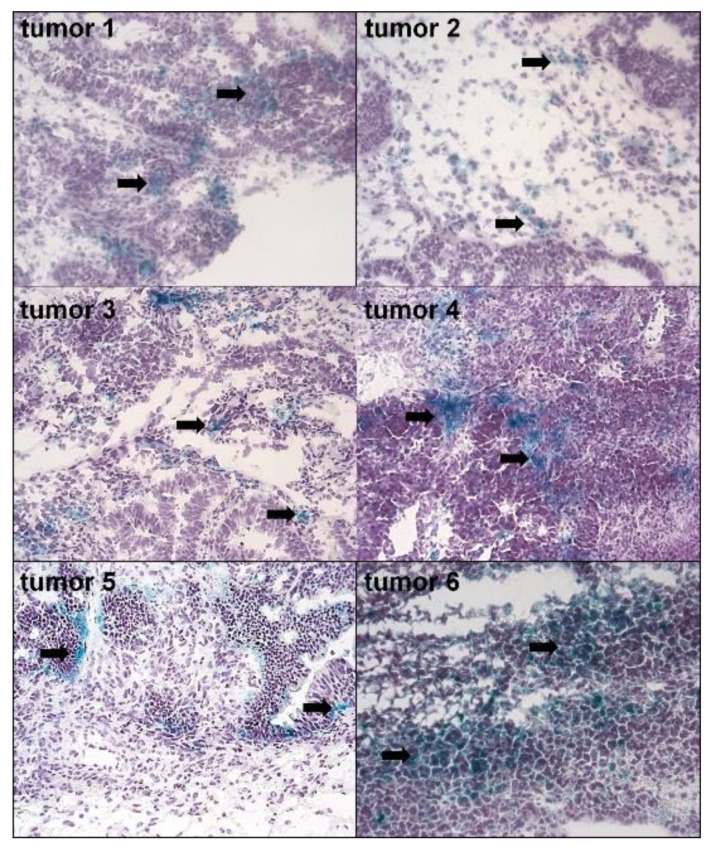
The presence of senescent cancer cells in ovarian tumors from patients who did not receive chemotherapy. Senescent cells were visualized according to the expression of SA-β-Gal (cells with green cytoplasm, marked with black arrows). The figure shows SA-β-Gal staining in six tumors out of the eleven investigated (magn. ×50).

Histochemical analysis of tumors excised from 11 pre-chemotherapy patients with pEOC revealed the abundance of senescent, SA-β-Gal-positive cells (Figure 1). However, the magnitude of specific, green staining of SA-β-Gal within the cytoplasm differed between patients. In some samples, senescent cells appeared incidentally (e.g., Tumors 2 and 3), whereas in others (e.g., Tumors 4 and 6) a considerable fraction of cancer cells were SA-β-Gal-positive. A quantitative analysis of the specimens (20 random fields per tumor) revealed that 34% ± 18% of the region of interest within the tested tumors, encompassing exclusively cancer cells, were positive for SA-β-Gal.

**Figure 2:**
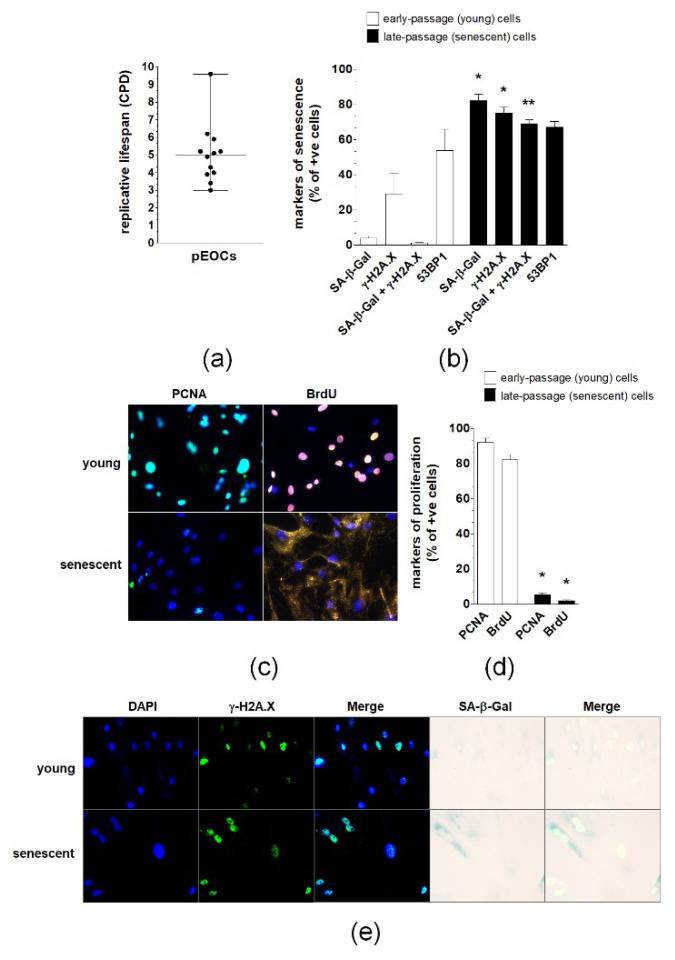
Replicative lifespan and expression of senescence biomarkers in primary epithelial ovarian cancer cells (pEOCs). (**a**) The replicative lifespan of the cells was estimated according to the cumulative number of population doublings (CPD). (**b**,**e**) Quantification of SA-β-Gal, γ-H2A.X, combined expression of SA-β-Gal and γ-H2A.X, and 53BP1 in early-passage and senescent pEOCs. (**c**,**d**) Analysis of early-passage and senescent pEOC proliferation according to determination of PCNA level and BrdU incorporation. Results are based on 6–8 independent experiments using pEOCs obtained from different patients. Results are expressed as mean ± SEM. * *p* < 0.05; ** *p* < 0.01 vs. early-passage cells.

In vitro estimation of the replicative lifespan of pEOCs established form ovarian tumors revealed that the growth capacity of these cells is limited and that they become growth-arrested after approximately five population doublings. Strong disparities regarding the lifespan of cancer cells were present as some cultures were unable to divide after three replications, whereas others were capable of reaching nine divisions (Figure 2A). A subset of early-passage cells displayed features of senescence, including activity of SA-β-Gal and expression of γ-H2A.X and 53BP1 foci. The percentage of cells exhibiting SA-β-Gal and γ-H2A.X, but not 53BP1, increased significantly during consecutive passages, reaching values of 80% of the population (Figure 2B,E). Simultaneously, the cells became enlarged, flattened, and were often multinucleated. In contrast to early-passage cultures, which displayed vigorous proliferation (manifested by high incorporation of BrdU and expression of PCNA), only a marginal fraction (<10%) of late-passage, senescent cells retained their proliferative potential (Figure 2C,D).

### 2.2. Changes in Cell Cycle During Spontaneous Senescence of pEOCs

**Figure 3:**
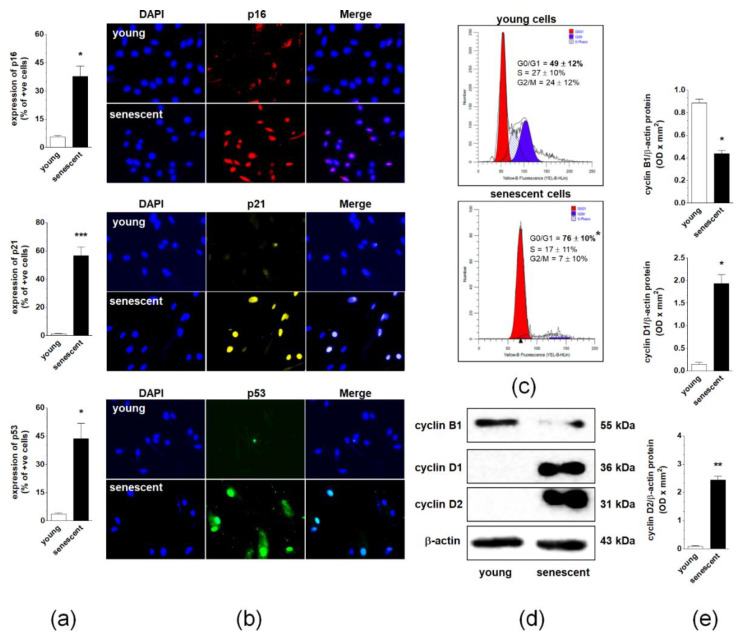
Regulation of pEOC senescence at the level of the cell cycle. (**a**,**b**) Quantification of p16, p21, and p53 cell cycle inhibitors in young and senescent pEOCs. (**c**) Histograms representing the distribution of young and senescent pEOCs in particular phases of the cell cycle. The cells in the G_1_ phase are marked in red, whereas those in the S phase are marked in blue. (**d**) Changes in cyclins B1, D1, and D2 levels in young and senescent pEOCs obtained using Western blot and quantified (**e**) with densitometry. Samples corresponding to 1 × 10^4^ cells were subjected to SDS–PAGE to eliminate the risk of incorrect results due to senescence-associated cell hypertrophy and related differences in protein content between young and senescent cells. Results are based on 6–8 independent experiments using pEOCs obtained from different patients. Results are expressed as mean ± SEM. * *p* < 0.05; ** *p* < 0.01; *** *p* < 0.001 vs. young cells.

Three cell cycle inhibitory proteins, p16, p21, and p53, were examined to identify effector pathways of spontaneous senescence in pEOCs. Immunofluorescence measurements showed that the replicative senescence of pEOCs is associated with significant up-regulation of these proteins. The sharpest increase was observed for p21, which was expressed in nearly 60% of late-passage cells. Positive staining for p16 and p53 was noted in approximately 40% and 45% of senescent cells, respectively (Figure 3A,B). Changes in the expression of cell cycle inhibitors were accompanied by growth arrest of late-passage cells in the G_1_ phase of the cell cycle. At the same time, the number of DNA-replicating cells in S phase markedly declined (Figure 3C). Flow cytometry analysis of the cell cycle was consistent with the analysis of cell cycle-promoting cyclins B1, D1, and D2. A semi-quantification of cyclins using immunoblotting showed a considerable decrease in the expression of the cyclin B1, and a simultaneous increase in the expression of cyclins D1 and D2 (Figure 3D,E).

### 2.3. Changes in Telomeres and Telomerase during Senescence of pEOCs

**Figure 4:**
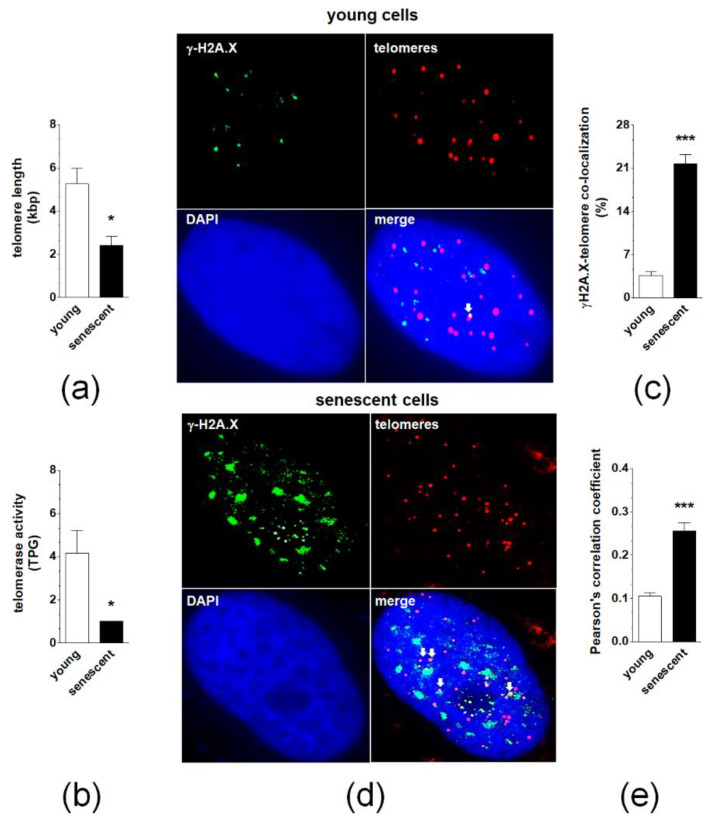
The role of telomeres and telomerase in spontaneous senescence of pEOCs. (**a**) Telomere length in young and senescent pEOCs according to qPCR. (**b**) Changes in telomerase activity during senescence of pEOCs based on hTERT quantification. (**c**,**d**) The magnitude of co-localization of histone γ-H2A.X (green) with telomeres (red) in young and senescent pEOCs. White arrows indicate places of co-localization (yellow). (**e**) Analysis of Pearson’s correlation coefficient between FITC and Cy3 images, showing the amount of co-localization between the two fluorescent images on a scale of +1 to −1, representing perfect co-localization to no co-localization, respectively. Results are based on 6–8 independent experiments using pEOCs obtained from different patients. Results are expressed as mean ± SEM. * *p* < 0.05; *** *p* < 0.001 vs. young cells. TPG—total product generated.

Quantitative PCR measuring telomere length revealed that senescence of pEOCs is associated with a significant deterioration of these structures (Figure 4A). This effect was accompanied by decreased activity of a catalytic subunit of telomerase, hTERT (Figure 4B). Analysis of individual γ-H2A.X-positive nuclei showed that in early-passage cells less than 10% of DNA damage foci co-localized to telomeres. In senescent cultures, the degree of co-localization significantly increased to 20–25% (Figure 4C,D). Quantitative examination of deconvoluted images, according to a Pearson’s correlation analysis, produced coefficients of 0.13 ± 0.03 and 0.25 ± 0.08 for young and senescent cells, respectively, confirming the relatively low degree of co-localization between γ-H2A.X foci and telomeres in both cases (Figure 4E).

### 2.4. Changes in Signaling Pathways Accompanying Senescence of pEOCs

**Figure 5:**
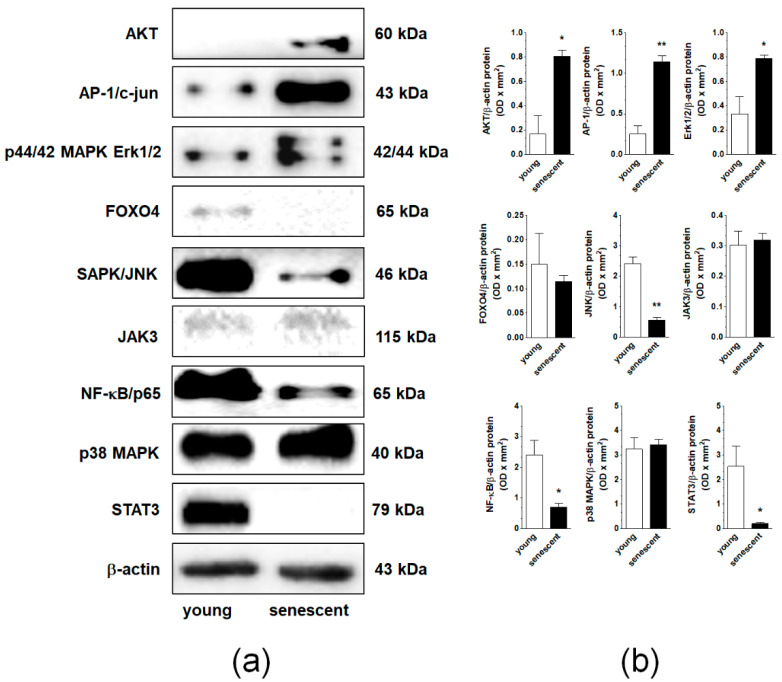
Changes in the expression of signaling molecules during senescence of pEOCs determined using immunoblotting. (**a**) Samples corresponding to 1 × 10^4^ (AKT, AP-1, ERK1/2, JNK, NF-κB, p38 MAPK, STAT3), 4 × 10^4^ (FOXO4, JAK3), and 5 × 10^4^ (β-actin) cells were subjected to SDS–PAGE to eliminate the risk of incorrect results due to senescence-associated cell hypertrophy and related differences in protein content between young and senescent cells. (**b**) Densitometric analysis of bands corresponding to young and senescent cells. Results are based on five to six independent experiments using pEOCs obtained from different patients. Results are expressed as mean ± SEM. * *p* < 0.05; ** *p* < 0.01 vs. early-passage cells.

Nine signaling molecules known to be engaged in cellular senescence, AKT, AP-1, ERK1/2, JNK, NF-κB, p38 MAPK, STAT3, FOXO4, and JAK3 were tested using Western blot in young and senescent pEOCs. Analysis revealed that the senescence of the cells was associated with an elevated expression of AKT, AP-1/c-jun, and p44/42 Erk1/2. Further, the expression level of SAPK/JNK, NF-κB/p65, and STAT3 was remarkably decreased. The expression of FOXO4, JAK3, and p38 MAPK remained unchanged (Figure 5).

### 2.5. Changes in Oxidative Stress-Related Parameters During Senescence of pEOCs

**Figure 6:**
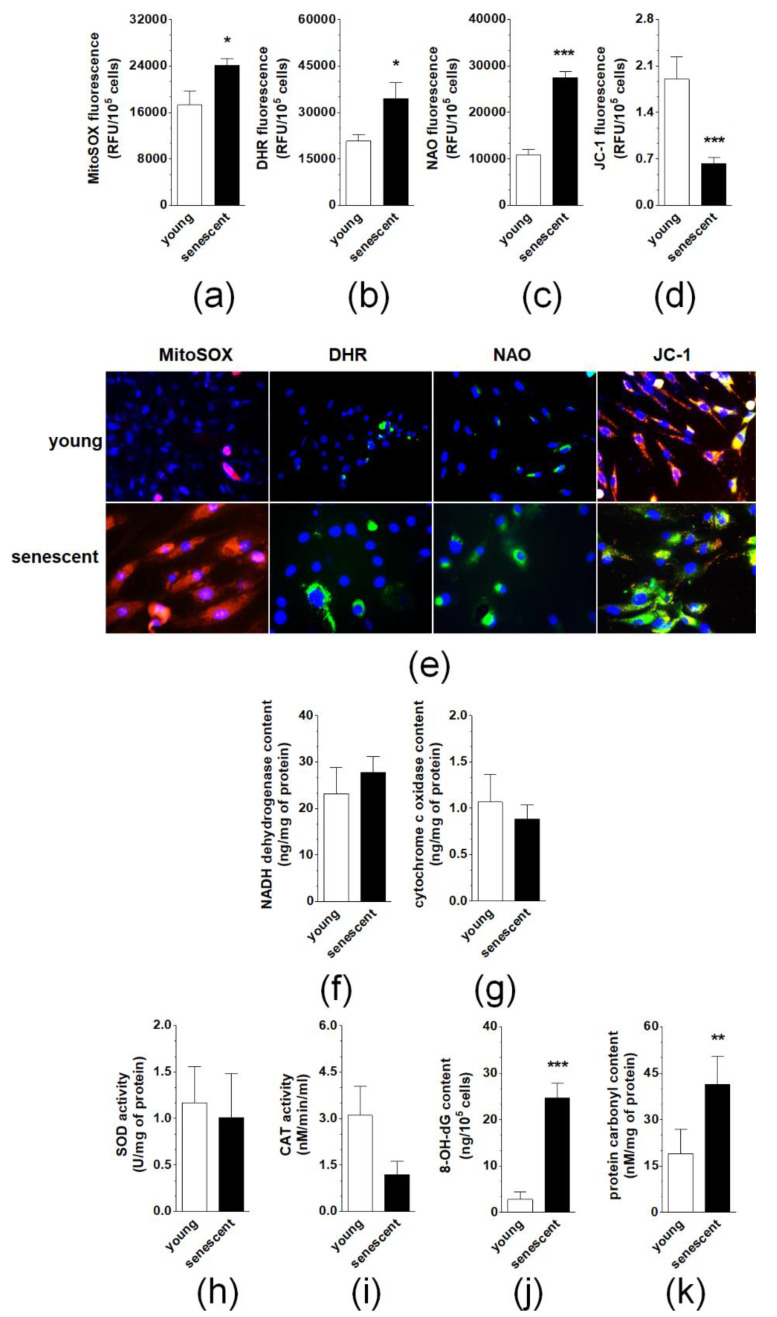
Analysis of oxidative stress-related parameters during pEOCs senescence. Changes in the production of (**a**) mitochondrial superoxides, (**b**) cellular peroxides, (**c**) mitochondrial mass, and (**d**) mitochondrial membrane potential in young and senescent pEOCs. (**e**) Representative pictures showing fluorescence of MitoSOX Red, DHR, NAO, and JC-1. Changes in the activity of (**f**) NADH dehydrogenase, (**g**) cytochrome c oxidase, (**h**) superoxide dismutase, (**i**) catalase in young and senescent pEOCs. Senescence-associated changes in the concentration of (**j**) 8-OH-dG and (**k**) carbonylated proteins. Results are based on eight independent experiments using pEOCs obtained from different patients. Results are expressed as mean ± SEM. * *p* < 0.05; ** *p* < 0.01; *** *p* < 0.001 vs. young cells.

Senescent pEOCs generate considerably higher amounts of mitochondrial superoxides and cellular peroxides than their young counterparts, as evidenced by fluorescent staining of cells with MitoSOX Red and DHR, respectively (Figure 6A,B,E). This effect coincided with enhanced mitochondrial mass (NAO fluorescence) and decreased ΔΨ_m_ potential (JC-1 fluorescence) (Figure 6C,D,E). Further, there was no change in the activity of NADH dehydrogenase or cytochrome c oxidase (Figure 6F,G). Similar lacks of statistically significant differences were observed upon comparison of young and senescent pEOCs with respect to the activity of two antioxidative enzymes, superoxide dismutase (total enzyme activity) and catalase (Figure 6H,I). Conversely, a significant, senescence-associated rise was found when concentrations of 8-OH-dG and carbonylated proteins were compared in young and senescent cells (Figure 6J,K).

### 2.6. Induction of pEOC Senescence by Normal Peritoneal Mesothelial Cells and Fibroblasts

**Figure 7:**
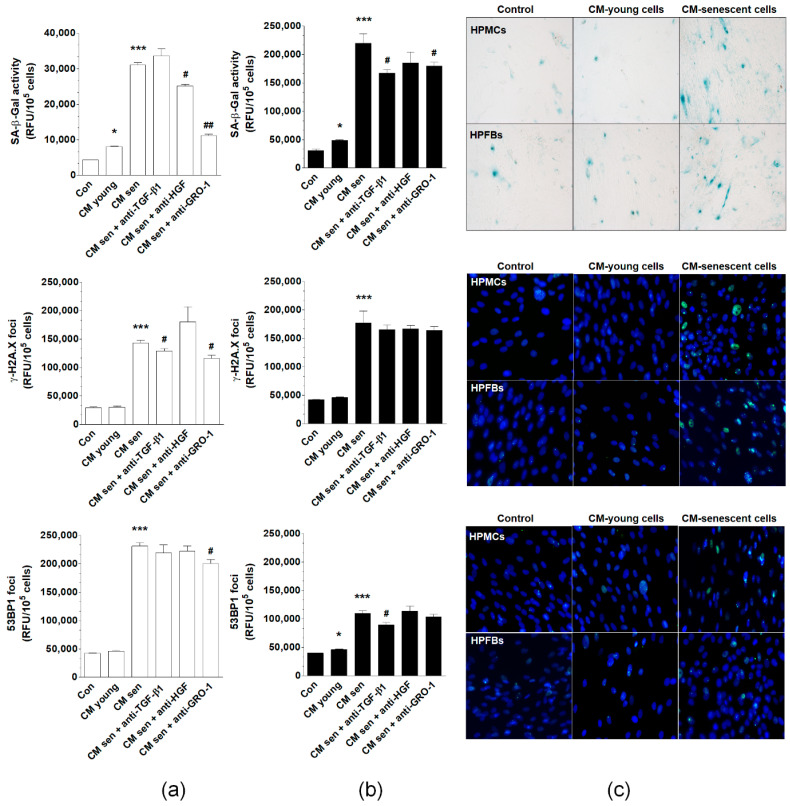
Effect of normal peritoneal mesothelial cells (HPMCs) and fibroblasts (HPFBs) on senescence induction in pEOCs. (**a**) Changes in SA-β-Gal, γ-H2A.X, and 53BP1 levels in pEOCs upon exposure to conditioned medium (CM) from young and senescent (**a**) HPMCs and (**b**) HPFBs. (**c**) Representative staining of SA-β-Gal, γ-H2A.X, and 53BP1 in pEOCs subjected to CM generated by HPMCs and HPFBs. Results are based on six independent experiments using pEOCs obtained from different patients. Results are expressed as mean ± SEM. * *p* < 0.05; *** *p* < 0.001 vs. Con.; # *p* < 0.05; ## *p* < 0.01. vs. CM from senescent cells. RFU—relative fluorescence units.

Primary omental mesothelial cells (HPMCs) and fibroblasts (HPFBs) were maintained in standard culture conditions and then conditioned medium (CM) was collected from young and senescent cells. CM samples were then applied to the pEOCs, followed by the evaluation of senescent phenotype development in these cells. The senescent phenotype was represented by SA-β-Gal activity and expression of γ-H2A.X and 53BP1 foci. Experiments using CM from HPMCs revealed that medium from young cells induces SA-β-Gal more strongly than when pEOCs were subjected to standard growth medium. There was no significant activation of γ-H2A.X and 53BP1 foci. At the same time, CM generated by senescent HPMCs yielded a significant up-regulation of SA-β-Gal, γ-H2A.X, and 53BP1 compared to pEOCs exposed to the CM from young cells. Co-culture with the CM from HPFBs yielded an effective induction of SA-β-Gal and 53BP1, but failed to modify γ-H2A.X. In addition, CM harvested from senescent HPFBs significantly increased all three tested biomarkers of senescence (Figure 7).

To identify possible soluble CM-derived mediators of the pEOC senescence, samples of CM from senescent HPMCs were preincubated with antibodies neutralizing GRO-1, HGF, and TGF-β1. Results showed that the activity of SA-β-Gal may be significantly reduced upon inactivation of GRO-1 and HGF, but not TGF-β1. The expression of γ-H2A.X foci was reduced upon neutralization of GRO-1 and TGF-β1, while the expression of the 53BP1 foci was decreased solely upon neutralization of GRO-1 (Figure 7A). When the same procedure was repeated for HPFB-derived CM, the stimulation of SA-β-Gal was abolished by inhibition of GRO-1 and TGF-β1, while the stimulation of 53BP1 was prevented via neutralization of TGF-β1. None of the neutralizations reduced the aforementioned senescent cell CM-dependent increase of γ-H2A.X (Figure 7A,B).

### 2.7. Induction of pEOC Senescence by Malignant Ascites

**Figure 8:**
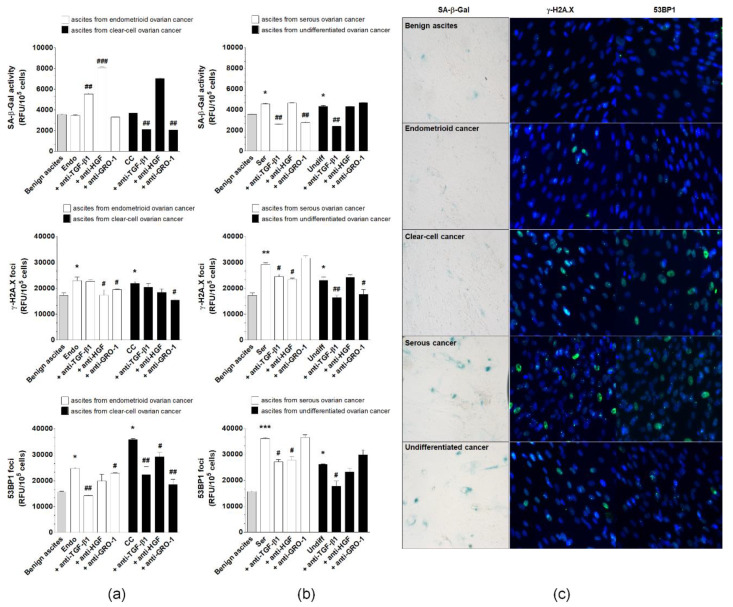
Effect of malignant ascites obtained from patients with endometrioid (Endo), clear cell (CC), serous (Ser), and undifferentiated (Undiff) ovarian cancer histotypes on senescence induction in pEOCs. Benign ascites were used as control. Changes in SA-β-Gal, γ-H2A.X, and 53BP1 levels in pEOCs upon exposure to malignant ascites generated by (**a**) endometrioid and clear-cell tumors, and (**b**) serous and undifferentiated tumors. (**c**) Representative staining of SA-β-Gal, γ-H2A.X, and 53BP1 in pEOCs subjected to benign and malignant ascites. Results are based on six independent experiments using pEOCs obtained from different patients. Results are expressed as mean ± SEM. * *p* < 0.05; ** p<0.01; *** *p* < 0.001 vs. benign ascites.; ^#^ *p* < 0.05; ^##^ *p* < 0.01; ^###^ *p* < 0.001 vs. Endo/CC/Ser/Undiff. RFU—relative fluorescence units.

Malignant ascites from patients with four EOC histotypes, endometrioid, clear cell, serous, and undifferentiated, were collected to assess their effect on pEOC senescence. Benign ascites were used as a control. pEOCs displayed increased activity of SA-β-Gal upon exposure to serous and undifferentiated ascitic fluid, but not by ascites from endometrioid or clear cell cancers. All malignant ascites effectively stimulated the formation of γ-H2A.X and 53BP1 foci (Figure 8).

To identify plausible, ascites-derived mediators of senescence, the fluids were incubated with neutralizing antibodies for GRO-1, HGF, and TGF-β1. The up-regulation of SA-β-Gal by serous malignant ascites was prevented by the inhibition of GRO-1 and TGF-β1, while the reaction resulting from the activity of ascites from undifferentiated tumors was only reduced in response to the neutralization of TGF-β1. Expression of γ-H2A.X foci, which was elevated after exposure to ascites from endometrioid tumors, was reduced by the inhibition of HGF. In the case of clear cell cancers, the expression of γ-H2A.X was suppressed upon neutralization of GRO-1, while ascites produced by serous and undifferentiated cancers led to the inhibition of histone γ-H2A.X foci formation after their incubation with antibodies against HGF-TGF-β1 and GRO-1-TGF-β1. The accumulation of 53BP1 foci in response to ascites from endometrioid cancers was also prevented by the neutralization of GRO-1-TGF-β1, and analysis of cells exposed to ascites from clear cell tumors revealed that an analogical effect was achievable with the inhibition of all tested proteins. Elevated 53BP1 fluorescence in cells treated with malignant ascites from serous and undifferentiated cancers was abolished when the fluid was preincubated with antibodies neutralizing HGF-TGF-β1 and TGF-β1 (Figure 8).

## 3. Discussion

To our knowledge, this is the first comprehensive analysis of both the phenomenology and mechanism of spontaneous senescence of primary epithelial ovarian cancer cells (pEOCs). The core of this study was the observation that all ovarian tumors obtained during cytoreduction from chemotherapy naïve patients contain a fraction of SA-β-Gal-positive, senescent cells [8]. The magnitude of this phenomenon is higher than in breast cancer patients, in whom only 10% of tumors expressed SA-β-Gal. In ovarian tumors, the senescent cells formed visible patches, while in breast tumors, only individual cells were identified as senescent [4]. Apart from breast cancer, the presence of spontaneously senescent cells in vivo has also been observed in mice with ductal hyperplasia and carcinoma in situ [9], as well as in Reed–Sternberg cells within Hodgkin’s lymphoma specimens [10].

These in vivo observations of the abundance of spontaneously senescent pEOCs agree with in vitro experiments on primary, early-passage cultures established from ovarian tumors, which revealed that cells exhibit biochemical (SA-β-Gal) and molecular (γ-H2A.X/53BP1) [11] signatures of cellular senescence. It is plausible that these cells appeared in culture conditions as a result of their direct transfer from the tumor mass during enzymatic disaggregation, albeit the sudden senescence syndrome typical for normal primary cell cultures [12] should not be dismissed. Nonetheless, the presence of the spontaneously senescent pEOCs in early-passage cultures resembles findings from primary prostate, breast, and colon cancer cells, which consistently exhibited features of spontaneous senescence [5]. The size of the senescent pEOC fraction is, however, smaller compared with other cancers. The percentage of cells bearing two standard markers of senescence, SA-β-Gal and γ-H2A.X, was below 10% compared to the 80%–90% observed in other primary cell cultures. The values recorded for pEOCs were more aligned with those reported for MDA-MB-231 breast cancer cells [13] and fibrosarcoma cells [14], which also showed enlarged morphology and positive staining for SA-β-Gal in 1%–3% of cells. Currently, one may only speculate that the large fraction of spontaneously senescent prostate, breast, and colon cancer cells could be a result of low efficiency of cell isolation, which translated to a high number of divisions passed by the cells before the formation of primary culture.

Interestingly, pEOCs entered senescence after relatively few divisions, suggesting poor proliferative potential and high vulnerability to environmental insult (culture shock) [15]. This behavior resembles ovarian cancer cells isolated from malignant ascites, which began to senesce between the 2nd and 8th passages [16]. Alternatively, the proliferative lifespan of pEOCs differs from p53-positive glioblastoma cells whose senescence was observed from the 15th to 20th passages onwards [17]. It should be stressed here that the proliferative capacity of pEOCs was homogenous, although the cultures were established from tumors belonging to four different histotypes, including aggressive high-grade serous and undifferentiated cancers and far less aggressive clear cell and endometrioid tumors [18]. The same conclusion was provided by authors of the research on ascites-derived ovarian cancer cells, in which no correlation between growth rate, histological subtype, or stage of disease at presentation was documented [16].

Phenomenological considerations of the spontaneous senescence of pEOCs were followed by attempts to identify a molecular background of this process. Senescent cells were found to be growth-arrested in the G_1_ phase of the cell cycle, the stage where the majority of normal cells undergo replicative senescence [19]. Colon cancer cells treated with high doses of DNA topoisomerase I inhibitor, SN-38 [4], and ovarian cancer cells exposed to poly(ADP-ribose) polymerase inhibitor, olaparib [20], behaved similarly. G_1_ growth-arrest was confirmed by specific changes in cyclins, particularly declined expression of cyclin B1 and increased expression of cyclins D1 and D2. As per cell cycle inhibitors, senescent pEOCs displayed upregulated expression of p16, p21, and p53. These findings are consistent with the report on senescent Hodgkin’s lymphoma-derived Reed–Sternberg cells [10] and epithelial cells overexpressing BRAF^V600E^ [21], which exhibited increased levels of p16 and p21. High levels of p16 were also observed in the spontaneous senescence of pre-malignant lesions in the lungs from K-*ras* V12 mice [22]. Up-regulation of p21 and p53 were found, in turn, in pre-neoplastic prostates of mice [23].

Interestingly, the highest senescence-associated increase in cell cycle inhibitors was recognized in the case of p21, which is known to contribute to, either independently or in conjunction with p53, DNA damage response-driven telomere-dependent senescence [24]. This mechanism of senescence in pEOCs is supported by increased co-localization of DNA damage foci (γ-H2A.X) with telomeres and significant shortening of telomeric DNA in senescent cells, possibly due to decreased telomerase activity [25]. At the same time, the majority of DNA damage foci were localized in non-telomeric regions of the genome, which, in combination with the short replicative lifespan of pEOCs and upregulated expression of p16, may imply that a considerable fraction of cells enters senescence in a telomere-independent manner [26]. Such a mosaic-like pattern of senescence has been shown in normal somatic cells including peritoneal mesothelium [27] and fibroblasts [28]. An intriguing shift in senescence effectors, which may also occur in pEOCs, was described in a separate type of ovarian cancer cells where a blockade of p21, acting as the initial mediator of senescence, was followed by compensatory up-regulation of p16 [29].

Diverse mechanisms of pEOC senescence translate to a specific pattern of changes in signaling molecules linked with senescence [30]. Senescent pEOCs display elevated levels of AKT, ERK1/2, and AP-1/c-jun, decreased expression of JNK, NF-κB, and STAT3, and unaltered p38 MAPK, JAK3, and FOXO4 signaling. As per the upregulated pathways, PI3K/AKT was found to induce senescence in immortalized human fibroblasts [31]. Similar activation has also been linked to the development of premature senescence upon activation of *Ras* or oxidative stress. AKT mediates senescence by increasing the production of reactive oxygen species (ROS), which is particularly important in the context of increased formation of ROS by senescent pEOCs [32]. For ERK1/2 and downstream transcription factor, activating protein-1 (AP-1) [33], a more complicated role in senescence is plausible. In normal somatic cells, ERK1/2 is essential for the transition from G_1_ to S phase of the cell cycle. At the same time, in cells expressing Ras or Raf mutants, ERK1/2 hyperactivation results in growth arrest [34]. Recently, the prolonged induction of ERK1/2 was found to contribute to the development of a senescence phenotype in HeLa cells [35]. Surprisingly, AP-1, which is generally characterized as pro-mitotic, has also been reported as increased in senescent pEOCs [36]. Recently, however, the ROS-mediated induction of AP-1 has been causatively connected with the initiation of senescence in endothelial cells [37]. AP-1 induction has also been suggested as an essential element of signaling cascade in *Ras*-induced senescence of fibroblasts [38].

Following the assessment of the phenotypic and mechanistic aspects of spontaneous senescence in pEOCs, the analysis of potential inducers of this phenomenon was performed. First, the research addressed the most common culprit of senescence, oxidative stress [39]. Senescent pEOCs overproduce both mitochondrial superoxides and cellular peroxides, ROS, which did not appear to be compensated by increased activity of the major antioxidants: superoxide dismutase and catalase. This imbalance leads to the accumulation of oxidatively modified DNA and proteins and may explain the erosion of telomeres in senescent cells [40]. Lack of changes in the activity of NADH dehydrogenase and cytochrome c oxidase implies that increased formation of mitochondrial ROS is not the result of leakage from the respiratory chain [41], but rather of increased biogenesis of the mitochondria in response to declining values of inner membrane potential (ΔΨ_m_) and an inability to sufficiently provide ATP. This reaction paradoxically leads to exacerbation of oxidative stress and is called retrograde-signaling. This is a common adaptation of mitochondria in replicatively senescent normal somatic cells [42].

Since cellular senescence, apart from the endogenous ROS induction, may also be induced by extracellular stressors [43], we examined if the senescence phenotype in pEOCs, marked by the presence of SA-β-Gal and γ-H2A.X/53BP1 foci, may be elicited in a paracrine manner by proteins released to environment (conditioned medium) by normal peritoneal mesothelial cells or fibroblasts, or present in malignant ascites. pEOCs maintained under such conditions were sensitive to the pro-senescence impact of the normal cells, especially their senescent forms, and malignant ascites. Mechanistically, this was associated with the activity of GRO-1, HGF, and TGF-β1. The observation that senescent HPMCs may initiate senescence in pEOCs is in opposition to our earlier findings, where we demonstrated using both in vitro and in vivo models, that senescent cells promote critical elements of cancer cell expansion, including adhesion [44], proliferation, migration, invasion [7], and angiogenesis [45]. It is noteworthy, however, that the influence of senescent HPMCs was found exclusively in the established, immortal A2780, OVCAR-3 and SKOV-3 cells, which, due to their genetic aberrations, are unable to become senescent. As per the pro-senescence effect of malignant ascites, similar activity was recently described in HPMCs, whose accelerated senescence translated to the increased progression of cancer cells [43]. Proteins recognized as mediators of paracrine senescence in pEOCs were previously found to exert a similar effect in fibroblasts (GRO-1 [46]), neuronal progenitors (GRO-1 [47]), peritoneal mesothelial cells (TGF-β1 [48]), hepatocellular cancer cells (TGF-β1 [49]), and lung adenocarcinoma cells [50]. Particularly intriguing activity was recently observed in ovarian cancer cell-derived HGF, which appeared capable of inducing accelerated senescence in HPMCs, actively contributing to the formation of a metastatic niche by these cells within the peritoneum [51]. It is also possible that exogenous pro-senescence stimuli may interfere with intracellular senescence machinery, as evidenced by TGF-β1, which has the ability to generate ROS [52], suppress hTERT [53], reduce the efficiency of DNA repair mechanisms [54], and deteriorate telomeres [55].

## 4. Materials and Methods

### 4.1. Chemicals

Unless otherwise stated, all chemicals were from Merck (Darmstadt, Germany). Plastics and other consumables were from Nunc (Roskilde, Denmark). Antibodies against HGF (# MAB294), GRO-1 (# MAB275R), and TGF-β1 (# AF-101-NA) were from R&D Systems (Abingdon, UK).

### 4.2. Tumors and Malignant Ascites

The study included tumors obtained from 48 women with ovarian cancer who were undergoing cytoreductive surgery. The tumors represented four histotypes: high-grade serous (*n* = 41), undifferentiated (*n* = 1), endometrioid (*n* = 2), and clear-cell (*n* = 4). The majority of the tumors were stage III and IV according to the criteria of the International Federation of Gynecology and Obstetrics. The age of the donors ranged from 41 to 83 years old. Cancerous tissue was identified using standard H+E staining. A part of each tumor (*n* = 11) was frozen and cut into 4 μM sections using the cryostat HM 525NX (ThermoFisher Scientific, Waltham, MA, USA). Remaining tumors were used to establish primary cell cultures. Planimetric analysis of a green-stained area reflecting the presence of senescent cells was quantified using ImageJ v1.52a software (http://rsb.info.nih.gov/ij/). Twenty × 100 fields per tumor were examined. The results were expressed as a percentage (%), and the whole area of a specimen was treated as 100%.

Malignant ascites were obtained from 32 patients with high-grade serous *(n* = 8), undifferentiated (*n* = 8), endometrioid (*n* = 8), and clear-cell cancer (*n* = 8). Benign fluids were obtained from age-matched patients with *cystadenoma mucinosum multiloculare* (*n* = 8). After collection in sterile conditions, the fluids were centrifuged at 2500 rpm for 10 min and the acellular supernatants were stored at −20 °C. The study was approved by an institutional ethics committee (consent number 578/18) and all patients gave their informed consent.

### 4.3. Cell Cultures

Primary epithelial ovarian cancer cells (pEOCs) were isolated from tumors obtained during cytoreductive surgery. The tumors were cut into small pieces of similar weight and placed into a solution of 0.05% trypsin and 0.02% EDTA for 20 min at 37 °C with gentle shaking. After resuspension in RPMI1640 containing 20% FBS, the cells were probed with an antibody directed against the epithelial-related antigen (MOC-31) and CA125 (both from Abcam, Cambridge, UK) to confirm their epithelial cancerous nature. Finally, ovarian cancer cells were cultured in RPMI 1640 supplemented with L-glutamine (2 mM) and 20% FBS. Replicative senescence of cancer cells was induced by serial passaging at fixed seeding density and time intervals until complete exhaustion of proliferative capacity. Senescence was confirmed according to the growth cessation combined with enlarged/flattened morphology and positive reaction for SA-β-Gal in the overwhelming majority of cells in culture. At each passage, cells were counted to estimate the cumulative number of population doublings (CPD).

Primary human peritoneal mesothelial cells (HPMCs) and peritoneal fibroblasts (HPFBs) were isolated by enzymatic digestion of the omentum, obtained from 10 female patients undergoing abdominal surgery. The age of the donors ranged from 51 to 86 years old. The study was approved by an institutional ethics committee (consent number 578/18) and all patients gave their informed consent. The HPMCs were grown in M199 medium with L-glutamine (2 mM), penicillin (100 U/mL), streptomycin (100 µg/mL), hydrocortisone (0.4 µg/mL) and 10% FBS. The HPFBs were maintained in Ham’s Nutrient Mixture F-12 medium enriched in the same supplements as for HPMCs. Both HPMCs and HPFBs were forced to replicative senescence using the same protocol as described for pEOCs.

In some experiments, early-passage pEOCs were exposed to 25% conditioned medium generated by young and senescent HPMCs and HPFBs, and to 10% benign and malignant ascites (for 72 h), in the presence (preincubation for 72 h) or absence of neutralizing antibodies against TGF-β1 (400 ng/mL), HGF (1 µg/mL), and GRO-1 (8.84 µg/mL).

### 4.4. Biomarkers of Senescence

In tumor sections, senescent cancer cells were identified according to a cytochemical visualization of SA-β-Gal, as described in [56]. The same method was used to detect senescent cancer cells in culture. Moreover, the acquisition of a senescent phenotype was verified using a fluorescence-based detection of senescence-associated DNA damage response (DDR) elements, i.e., the foci of the phosphorylated variant of histone H2A.X (γ-H2A.X) and p53-binding protein 1 (53BP1). Both of the DDR proteins were examined using a method described in detail in [51].

In some experiments, the immunofluorescence for γ-H2A.X was followed by the detection of telomeric ends using the Telomere PNA FISH Kit/Cy3 (Dako, Carpinteria, CA, USA), as per the manufacturer’s instructions. For co-localization studies, fluorescent images were deconvoluted and analyzed using the Costes approximation method in ImageJ v1.52a software (http://rsb.info.nih.gov/ij/), with the plugin bundle from the Wright Cell Imaging Facility (http://www.uhnresearch.ca/facilities/wcif/imagej/). For each nucleus, a Pearson’s correlation coefficient (R) was determined.

### 4.5. Proliferating Cell Nuclear Antigen (PCNA)

PCNA expression was detected using immunofluorescence. The cells were fixed with 4% paraformaldehyde (PFA) for 10 min, permeabilized with 0.3% Triton X-100 in phosphate-buffered saline (PBS) for 10 min, and blocked with 5% goat serum and 0.3% Triton X-100 in PBS for 60 min. Afterwards, the cells were incubated overnight at 4 °C with Alexa Fluor 488 conjugated anti-PCNA mouse monoclonal antibody (#8580, Cell Signaling Technology, Danvers, MA, USA), diluted 1:100. The nuclei were stained with DAPI (#ab104139, Abcam, Cambridge, UK).

### 4.6. Bromodeoxyuridine (BrdU) Uptake

In order to examine the BrdU uptake, cells were incubated with 10 µM BrdU for 72 h at 37 °C. Cells were then fixed with 4% PFA for 15 min at room temperature, permeabilized with 0.1% Triton X-100 in PBS (for 20 min at room temperature), incubated with 1N HCl (for 10 min on ice), treated with 2N HCl (for 10 min at room temperature), and incubated with phosphate/citric acid buffer, pH 7.4 (for 10 min at room temperature). Afterwards, the cells were incubated with 10 µg/mL anti-BrdU antibody (#MAB7225, R&D Systems, Abingdon, UK) in 0.1% Triton X-100/5% goat serum in PBS for 24 h at room temperature. After incubation, cells were rinsed with a permeabilization buffer and stained using Alexa Fluor 555 goat anti-mouse IgG (#A32727, Invitrogen, Carlsbad, CA, USA), diluted at 1:500 for 1 h at room temperature. The nuclei were stained with DAPI.

### 4.7. Cell Cycle Distribution and Expression of Cell Cycle Inhibitory Proteins

Cell cycle distribution was quantified using the GUAVA EasyCyte 6HT-2L flow cytometer (Merck) with ModFit LT™ software (Verity Software House, Topsham, ME, USA), as described in [57]. Expression of p16, p21, and p53 cell cycle inhibitory proteins was detected using immunofluorescence. To this end, cells were fixed with 4% PFA (for 10 min at room temperature) and 100% methanol (for 5 min at −20 °C), permeabilized with 0.1% Triton-X in PBS for 10 min, and blocked with 1% bovine serum albumin, 22.52 mg/mL glycine, and 0.1% Triton-X in PBS for 30 min. Afterwards, the cells were incubated with a rabbit antibody against human p16 (#ab108349; Abcam), p21 (#2947, Cell Signaling), and p53 (#2527, Cell Signaling), all diluted 1:200, for 24 h, at 4 °C. Next, the cells were washed and treated with DyLight 488 polyclonal goat anti-rabbit IgG (#ab96899, Abcam) diluted 1:500 for 1.5 h at room temperature. After the incubation, specimens were mounted with a fluoroshield medium containing DAPI (Abcam) and inspected under a fluorescent microscope.

### 4.8. Cyclins and Signaling Pathways

Expression of cyclins B1, D1, and D2 was examined using immunoblotting. Cancer cells were lysed in a buffer containing 10 mM Tris-HCl at pH 7.5, 150 mM NaCl (BioShop Canada Inc, Burlington, ON, Canada), 0.1% SDS, 1% IGEPAL CA-630^®^, 0.5% deoxycholic acid (BioShop Canada Inc.), and a protease inhibitor cocktail (PIC003.1 BioShop Canada Inc.). Samples corresponding to an equal, experimentally optimized number of cells were subjected to SDS–PAGE. Afterwards, the proteins were transferred to a PVDF membrane with 0.22 µm pores (Serva, Heidelberg, Germany) by semi-dry transfer using the Trans Blot^®^ Turbo Blotting System (Bio-Rad, Hercules, CA, USA) under 0.30 A for 30 min or wet transfer using the Mini Trans-Blot^®^ Module (Bio-Rad) under 19 V, overnight at 4 °C. The membranes were immunoblotted with rabbit antibody against AKT (#9272), cyclin B1 (#12231), cyclin D1 (#2978), cyclin D2 (#3741), FOXO4 (#9472), JAK3 (#8863), NF-κB p65 (#8242), p38 MAPK (#9212), p44/42 MAPK Erk1/2 (#4695), SAPK/JNK (#9252), STAT3 (#12640) (all from Cell Signaling Technology, diluted 1:1000) and specific primary mouse antibody against β-actin as the control (#sc-1616 Santa-Cruz, Dallas, TX, USA). Bound antibodies were visualized following incubation with the peroxidase-labeled secondary antibodies (Cell Signaling), diluted 1:2000, for 1 h, at room temperature, and exposed using the Western Bright^™^ Sirius HRP substrate (Advansta, San Jose, CA, USA) for 2 min at room temperature, in the dark. The whole blot figures are provided in the Supplementary Materials.

### 4.9. Telomere Length and Telomerase Activity

Telomere length was measured using Absolute Human Telomere Length Quantification qPCR Assay Kit (ScienCell, Carlsbad, CA, USA). Genomic DNA was extracted using the Genomic Mini Kit (A&A Biotechnology, Gdynia, Poland). The PCR reaction was performed using a LightCycler 96 (Roche, Penzberg, Germany) in the presence of a FastStart Essential DNA Green Master (Roche) and 5 ng of DNA. The Comparative Quantification Cycle Value method was used for the calculation of telomere length.

Telomerase activity was assessed using the TRAPEZE XL telomerase detection kit (Merck), where the telomerase reaction products are amplified by PCR. The assay was performed in an ELISA format following the manufacturer’s instructions. The test was performed with 2 μg of cell lysate.

### 4.10. Oxidative Stress and Mitochondrial Metabolism

The generation of mitochondrial superoxides and cellular peroxides were monitored in cells probed with MitoSOX Red (Thermo Fisher Scientific, Waltham, MA, USA) and dihydrorhodamine 123 (DHR), respectively. Mitochondrial membrane potential (ΔΨ_m_) was quantified in cells probed with 5,5′,6,6′-tetrachloro-1,1′,3,3′-tetraethylbenzimidazolylcarbocyanine iodide (JC-1; Cayman Chemical), whereas mitochondrial mass was tested upon cell exposure to 10-*n*-nonyl-acridine orange (NAO). All procedures were performed as described in [58]. Activity of NADH dehydrogenase and cytochrome c oxidase were analyzed using commercial ELISA-based kits from Wuhan EIAab Science Co., Ltd. (Wuhan, China). Activity of superoxide dismutase (total) and catalase, as well as concentration carbonylated proteins were quantified using dedicated assays obtained from Cayman Chemical (Ann Arbor, MI, USA). Concentration of 8-hydroxy-2′-deoxyguanosine (8-OH-dG) was measured using DNA damage (8-OHdG) ELISA Kit, purchased from Biorbyt (Cambridge, UK). All commercial assays were performed according to the manufacturer’s instructions.

### 4.11. Statistics

Statistical analysis was conducted using GraphPad Prism™ 5.00 (GraphPad Software, San Diego, USA). The means were compared using the Wilcoxon signed-rank test. The results were expressed as mean ± SEM. Differences with a *p*-value < 0.05 were considered to be statistically significant.

## 5. Conclusions

In conclusion, our study suggests that the biology of pEOCs is more similar to normal cells than previously thought. Senescence of these cells is reminiscent of the replicative senescence of normal somatic cells in all aspects, including a finite replicative lifespan, a phenotype, changes at the level of cell cycle, telomeres and signaling molecules, the role of oxidative stress, and the propensity to be induced by extracellular stimuli. In this paper, we concentrated our attention on the mechanistic background of spontaneous senescence in pEOCs, and thus we did not address the biological and clinical relevance of this process. Further studies using in vitro and in vivo models are needed to answer the question of whether senescent pEOCs promote progression (e.g., proliferation, migration, invasion, epithelial-mesenchymal transition) of their young counterparts and/or angiogenic behavior of vascular endothelium similarly to senescent normal cells? And if so, whether this activity is underlined by the acquisition of senescence-associated secretory phenotype (SASP)? Other critical issues to be addressed include the effect of pEOCs senescence on the response to chemotherapy and the relation of this process to other outcomes of therapy, such as apoptotic cell death.

## Supplementary Materials

The following are available online at https://www.mdpi.com/2072-6694/12/2/296/s1, The whole immunoblotting membranes.

## References

[1] Childs B.G., Durik M., Baker D.J., van Deursen J.M. (2015). Cellular senescence in aging and age-related disease: From mechanisms to therapy. Nat. Med..

[2] He X., Yang A., McDonald D.G., Riemer E.C., Vanek K.N., Schulte B.A., Wang G.Y. (2017). MiR-34a modulates ionizing radiation-induced senescence in lung cancer cells. Oncotarget.

[3] Ewald J.A., Desotelle J.A., Wilding G., Jarrard D.F. (2010). Therapy-induced senescence in cancer. J. Natl. Cancer Inst..

[4] te Poele R.H., Okorokov A.L., Jardine L., Cummings J., Joel S.P. (2002). DNA damage is able to induce senescence in tumor cells in vitro and in vivo. Cancer Res..

[5] Zieba J., Ksiazkiewcz M., Janik K., Banaszczyk M., Peciak J., Piaskowski S., Lipinski M., Olczak M., Stoczynska-Fidelus E., Rieske P. (2015). Sensitivity of neoplastic cells to senescence unveiled under standard cell culture conditions. Anticancer Res..

[6] Ruhland M.K., Loza A.J., Capietto A.H., Luo X., Knolhoff B.L., Flanagan K.C., Belt B.A., Alspach E., Leahy K., Luo J. (2016). Stromal senescence establishes an immunosuppressive microenvironment that drives tumorigenesis. Nat. Commun..

[7] Mikula-Pietrasik J., Uruski P., Sosinska P., Maksin K., Piotrowska-Kempisty H., Kucinska M., Murias M., Szubert S., Wozniak A., Szpurek D. (2016). Senescent peritoneal mesothelium creates a niche for ovarian cancer metastases. Cell Death. Dis..

[8] Sosinska P., Mikula-Pietrasik J., Ryzek M., Naumowicz E., Ksiazek K. (2014). Specificity of cytochemical and fluorescence methods of senescence-associated beta-galactosidase detection for ageing driven by replication and time. Biogerontology.

[9] Miller F.R., Soule H.D., Tait L., Pauley R.J., Wolman S.R., Dawson P.J., Heppner G.H. (1993). Xenograft model of progressive human proliferative breast disease. J. Natl. Cancer Inst..

[10] Gopas J., Stern E., Zurgil U., Ozer J., Ben-Ari A., Shubinsky G., Braiman A., Sinay R., Ezratty J., Dronov V. (2016). Reed-Sternberg cells in Hodgkin’s lymphoma present features of cellular senescence. Cell Death Dis..

[11] d’Adda di Fagagna F., Reaper P.M., Clay-Farrace L., Fiegler H., Carr P., von Zglinicki T., Saretzki G., Carter N.P., Jackson S.P. (2003). A DNA damage checkpoint response in telomere-initiated senescence. Nature.

[12] Rubelj I., Huzak M., Brdar B. (2000). Sudden senescence syndrome plays a major role in cell culture proliferation. Mech. Ageing Dev..

[13] Cukusic A., Ivankovic M., Skrobot N., Ferenac M., Gotic I., Matijasic M., Polancec D., Rubelj I. (2006). Spontaneous senescence in the MDA-MB-231 cell line. Cell Prolif..

[14] Chang B.D., Broude E.V., Dokmanovic M., Zhu H., Ruth A., Xuan Y., Kandel E.S., Lausch E., Christov K., Roninson I.B. (1999). A senescence-like phenotype distinguishes tumor cells that undergo terminal proliferation arrest after exposure to anticancer agents. Cancer Res..

[15] Sherr C.J., DePinho R.A. (2000). Cellular senescence: Mitotic clock or culture shock?. Cell.

[16] O’Donnell R.L., McCormick A., Mukhopadhyay A., Woodhouse L.C., Moat M., Grundy A., Dixon M., Kaufman A., Soohoo S., Elattar A. (2014). The use of ovarian cancer cells from patients undergoing surgery to generate primary cultures capable of undergoing functional analysis. PLoS ONE.

[17] Stoczynska-Fidelus E., Piaskowski S., Bienkowski M., Banaszczyk M., Hulas-Bigoszewska K., Winiecka-Klimek M., Radomiak-Zaluska A., Och W., Borowiec M., Zieba J. (2014). The failure in the stabilization of glioblastoma-derived cell lines: Spontaneous in vitro senescence as the main culprit. PLoS ONE.

[18] Shih I., Kurman R.J. (2004). Ovarian tumorigenesis: A proposed model based on morphological and molecular genetic analysis. Am. J. Pathol..

[19] Ksiazek K., Piwocka K., Brzezinska A., Sikora E., Zabel M., Breborowicz A., Jorres A., Witowski J. (2006). Early loss of proliferative potential of human peritoneal mesothelial cells in culture: The role of p16INK4a-mediated premature senescence. J. Appl. Physiol..

[20] Wang Z., Gao J., Zhou J., Liu H., Xu C. (2019). Olaparib induced senescence under p16 or p53 dependent manner in ovarian cancer. J. Gynecol. Oncol..

[21] Zeppernick F., Ardighieri L., Hannibal C.G., Vang R., Junge J., Kjaer S.K., Zhang R., Kurman R.J., Shih I. (2014). BRAF mutation is associated with a specific cell type with features suggestive of senescence in ovarian serous borderline (atypical proliferative) tumors. Am. J. Surg. Pathol..

[22] Collado M., Gil J., Efeyan A., Guerra C., Schuhmacher A.J., Barradas M., Benguria A., Zaballos A., Flores J.M., Barbacid M. (2005). Tumour biology: Senescence in premalignant tumours. Nature.

[23] Chen Z., Trotman L.C., Shaffer D., Lin H.K., Dotan Z.A., Niki M., Koutcher J.A., Scher H.I., Ludwig T., Gerald W. (2005). Crucial role of p53-dependent cellular senescence in suppression of Pten-deficient tumorigenesis. Nature.

[24] Herbig U., Jobling W.A., Chen B.P., Chen D.J., Sedivy J.M. (2004). Telomere shortening triggers senescence of human cells through a pathway involving ATM, p53, and p21(CIP1), but not p16(INK4a). Mol. Cell.

[25] Passos J.F., Saretzki G., Ahmed S., Nelson G., Richter T., Peters H., Wappler I., Birket M.J., Harold G., Schaeuble K. (2007). Mitochondrial dysfunction accounts for the stochastic heterogeneity in telomere-dependent senescence. PLoS Biol..

[26] Ramirez R.D., Morales C.P., Herbert B.S., Rohde J.M., Passons C., Shay J.W., Wright W.E. (2001). Putative telomere-independent mechanisms of replicative aging reflect inadequate growth conditions. Genes Dev..

[27] Ksiazek K., Mikula-Pietrasik J., Olijslagers S., Jorres A., von Zglinicki T., Witowski J. (2009). Vulnerability to oxidative stress and different patterns of senescence in human peritoneal mesothelial cell strains. Am J Physiol. Regul. Integr. Comp. Physiol..

[28] Alcorta D.A., Xiong Y., Phelps D., Hannon G., Beach D., Barrett J.C. (1996). Involvement of the cyclin-dependent kinase inhibitor p16 (INK4a) in replicative senescence of normal human fibroblasts. Proc. Natl. Acad. Sci. USA.

[29] Diep C.H., Charles N.J., Gilks C.B., Kalloger S.E., Argenta P.A., Lange C.A. (2013). Progesterone receptors induce FOXO1-dependent senescence in ovarian cancer cells. Cell Cycle.

[30] Greer E.L., Brunet A. (2008). Signaling networks in aging. J Cell Sci..

[31] Astle M.V., Hannan K.M., Ng P.Y., Lee R.S., George A.J., Hsu A.K., Haupt Y., Hannan R.D., Pearson R.B. (2012). AKT induces senescence in human cells via mTORC1 and p53 in the absence of DNA damage: Implications for targeting mTOR during malignancy. Oncogene.

[32] Nogueira V., Park Y., Chen C.C., Xu P.Z., Chen M.L., Tonic I., Unterman T., Hay N. (2008). Akt determines replicative senescence and oxidative or oncogenic premature senescence and sensitizes cells to oxidative apoptosis. Cancer Cell.

[33] Glauser D.A., Schlegel W. (2007). Sequential actions of ERK1/2 on the AP-1 transcription factor allow temporal integration of metabolic signals in pancreatic beta cells. FASEB J..

[34] Meloche S., Pouyssegur J. (2007). The ERK1/2 mitogen-activated protein kinase pathway as a master regulator of the G1- to S-phase transition. Oncogene.

[35] Kudo I., Nozawa M., Miki K., Takauji Y., En A., Fujii M., Ayusawa D. (2016). Dual roles of ERK1/2 in cellular senescence induced by excess thymidine in HeLa cells. Exp. Cell Res..

[36] Shaulian E., Karin M. (2001). AP-1 in cell proliferation and survival. Oncogene.

[37] Olmos G., Martinez-Miguel P., Alcalde-Estevez E., Medrano D., Sosa P., Rodriguez-Manas L., Naves-Diaz M., Rodriguez-Puyol D., Ruiz-Torres M.P., Lopez-Ongil S. (2017). Hyperphosphatemia induces senescence in human endothelial cells by increasing endothelin-1 production. Aging Cell.

[38] Kwong J., Chen M., Lv D., Luo N., Su W., Xiang R., Sun P. (2013). Induction of p38delta expression plays an essential role in oncogenic ras-induced senescence. Mol. Cell Biol..

[39] Ksiazek K., Mikula-Pietrasik J., Jorres A., Witowski J. (2008). Oxidative stress-mediated early senescence contributes to the short replicative life span of human peritoneal mesothelial cells. Free Radic. Biol. Med..

[40] Von Zglinicki T., Pilger R., Sitte N. (2000). Accumulation of single-strand breaks is the major cause of telomere shortening in human fibroblasts. Free Radic. Biol. Med..

[41] Allen R.G., Tresini M., Keogh B.P., Doggett D.L., Cristofalo V.J. (1999). Differences in electron transport potential, antioxidant defenses, and oxidant generation in young and senescent fetal lung fibroblasts (WI-38). J. Cell Physiol..

[42] Jazwinski S.M. (2013). The retrograde response: When mitochondrial quality control is not enough. Biochim. Biophys. Acta.

[43] Mikula-Pietrasik J., Uruski P., Matuszkiewicz K., Szubert S., Moszynski R., Szpurek D., Sajdak S., Tykarski A., Ksiazek K. (2016). Ovarian cancer-derived ascitic fluids induce a senescence-dependent pro-cancerogenic phenotype in normal peritoneal mesothelial cells. Cell Oncol. (Dordr.).

[44] Ksiazek K., Mikula-Pietrasik J., Korybalska K., Dworacki G., Jorres A., Witowski J. (2009). Senescent peritoneal mesothelial cells promote ovarian cancer cell adhesion: The role of oxidative stress-induced fibronectin. Am. J. Pathol..

[45] Mikula-Pietrasik J., Sosinska P., Naumowicz E., Maksin K., Piotrowska H., Wozniak A., Szpurek D., Ksiazek K. (2016). Senescent peritoneal mesothelium induces a pro-angiogenic phenotype in ovarian cancer cells in vitro and in a mouse xenograft model in vivo. Clin. Exp. Metastasis.

[46] Yang G., Rosen D.G., Zhang Z., Bast R.C., Mills G.B., Colacino J.A., Mercado-Uribe I., Liu J. (2006). The chemokine growth-regulated oncogene 1 (Gro-1) links RAS signaling to the senescence of stromal fibroblasts and ovarian tumorigenesis. Proc. Natl. Acad. Sci. USA.

[47] Zonis S., Breunig J.J., Mamelak A., Wawrowsky K., Bresee C., Ginzburg N., Chesnokova V. (2018). Inflammation-induced Gro1 triggers senescence in neuronal progenitors: Effects of estradiol. J. Neuroinflammation.

[48] Ksiazek K., Korybalska K., Jorres A., Witowski J. (2007). Accelerated senescence of human peritoneal mesothelial cells exposed to high glucose: The role of TGF-beta1. Lab. Investig..

[49] Senturk S., Mumcuoglu M., Gursoy-Yuzugullu O., Cingoz B., Akcali K.C., Ozturk M. (2010). Transforming growth factor-beta induces senescence in hepatocellular carcinoma cells and inhibits tumor growth. Hepatology.

[50] Katakura Y., Nakata E., Miura T., Shirahata S. (1999). Transforming growth factor beta triggers two independent-senescence programs in cancer cells. Biochem. Biophys. Res. Commun..

[51] Mikula-Pietrasik J., Uruski P., Pakula M., Maksin K., Szubert S., Wozniak A., Naumowicz E., Szpurek D., Tykarski A., Ksiazek K. (2017). Oxidative stress contributes to hepatocyte growth factor-dependent pro-senescence activity of ovarian cancer cells. Free Radic. Biol. Med..

[52] Yoon Y.S., Lee J.H., Hwang S.C., Choi K.S., Yoon G. (2005). TGF beta1 induces prolonged mitochondrial ROS generation through decreased complex IV activity with senescent arrest in Mv1Lu cells. Oncogene.

[53] Li H., Xu D., Li J., Berndt M.C., Liu J.P. (2006). Transforming growth factor beta suppresses human telomerase reverse transcriptase (hTERT) by Smad3 interactions with c-Myc and the hTERT gene. J. Biol. Chem..

[54] Pal D., Pertot A., Shirole N.H., Yao Z., Anaparthy N., Garvin T., Cox H., Chang K., Rollins F., Kendall J. (2017). TGF-beta reduces DNA ds-break repair mechanisms to heighten genetic diversity and adaptability of CD44+/CD24- cancer cells. Elife.

[55] Deng W., Tsao S.W., Kwok Y.K., Wong E., Huang X.R., Liu S., Tsang C.M., Ngan H.Y., Cheung A.N., Lan H.Y. (2008). Transforming growth factor beta1 promotes chromosomal instability in human papillomavirus 16 E6E7-infected cervical epithelial cells. Cancer Res..

[56] Dimri G.P., Lee X., Basile G., Acosta M., Scott G., Roskelley C., Medrano E.E., Linskens M., Rubelj I., Pereira-Smith O. (1995). A biomarker that identifies senescent human cells in culture and in aging skin in vivo. Proc. Natl. Acad. Sci. USA.

[57] Mikula-Pietrasik J., Kuczmarska A., Rubis B., Filas V., Murias M., Zielinski P., Piwocka K., Ksiazek K. (2012). Resveratrol delays replicative senescence of human mesothelial cells via mobilization of antioxidative and DNA repair mechanisms. Free Radic. Biol. Med..

[58] Pakula M., Mikula-Pietrasik J., Stryczynski L., Uruski P., Szubert S., Moszynski R., Szpurek D., Sajdak S., Tykarski A., Ksiazek K. (2018). Mitochondria-related oxidative stress contributes to ovarian cancer-promoting activity of mesothelial cells subjected to malignant ascites. Int. J. Biochem. Cell Biol..

