# Correction: Pakuła et al. Deciphering the Molecular Mechanism of Spontaneous Senescence in Primary Epithelial Ovarian Cancer Cells. *Cancers* 2020, *12*, 296

**DOI:** 10.3390/cancers15030937

**Published:** 2023-02-02

**Authors:** Martyna Pakuła, Ewa Mały, Paweł Uruski, Anna Witucka, Małgorzata Bogucka, Natalia Jaroszewska, Nicoletta Makowska, Arkadiusz Niklas, Rafał Moszyński, Stefan Sajdak, Andrzej Tykarski, Justyna Mikuła-Pietrasik, Krzysztof Książek

**Affiliations:** 1 Department of Hypertensiology, Angiology and Internal Medicine, Poznan University of Medical Sciences, Długa 1/2 Str., 61-848 Poznan, Poland; 2 Poznan University of Medical Sciences Core Facility, Rokietnicka 8 Str., 60-806 Poznan, Poland; 3 Division of Gynecological Surgery, Poznan University of Medical Sciences, Polna 33 Str., 60-535 Poznan, Poland

## Error in Figures

In the original publication [1], a mistake was identified in Figure 5 and Figure 7. In Figure 5, new bands for FOXO4 and JAK3 proteins have been inserted that are consistent with the original uncut membranes included in the supplement. The results have been re-analyzed densitometrically, and new graphs for these proteins are included in panel (b). In Figure 7, one of the SA-β-Gal images that was mistakenly duplicated (control PFBs) has been replaced with the correct one.

The authors state that the scientific conclusions are unaffected. This correction was approved by the Academic Editor. The original publication has also been updated.

## Figures and Tables

**Figure 5:**
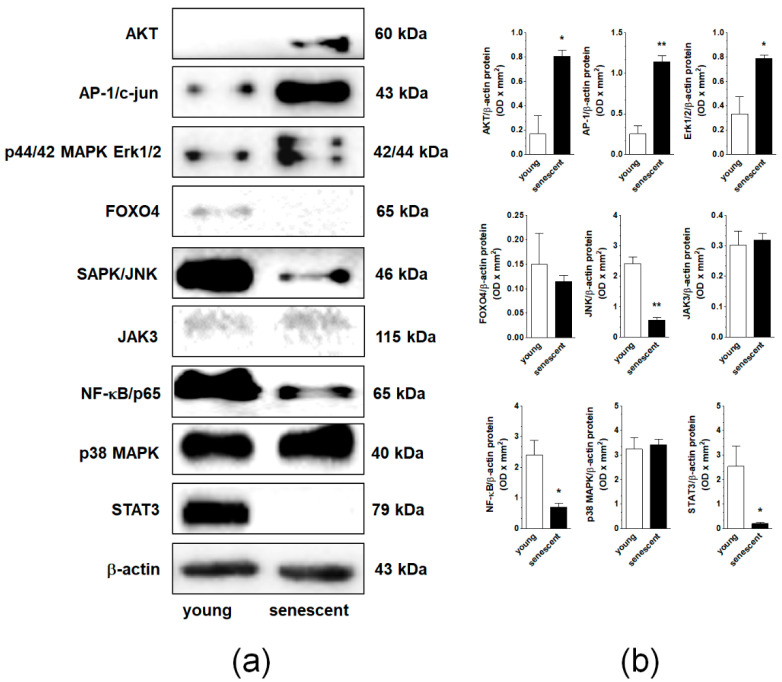
Changes in the expression of signaling molecules during senescence of pEOCs determined using immunoblotting. (**a**) Samples corresponding to 1 × 10^4^ (AKT, AP-1, ERK1/2, JNK, NF-κB, p38 MAPK, STAT3), 4 × 10^4^ (FOXO4, JAK3), and 5 × 10^4^ (β-actin) cells were subjected to SDS–PAGE to eliminate the risk of incorrect results due to senescence-associated cell hypertrophy and related differences in protein content between young and senescent cells. (**b**) Densitometric analysis of bands corresponding to young and senescent cells. Results are based on five to six independent experiments using pEOCs obtained from different patients. Results are expressed as mean ± SEM. * *p* < 0.05; ** *p* < 0.01 vs. early-passage cells.

**Figure 7:**
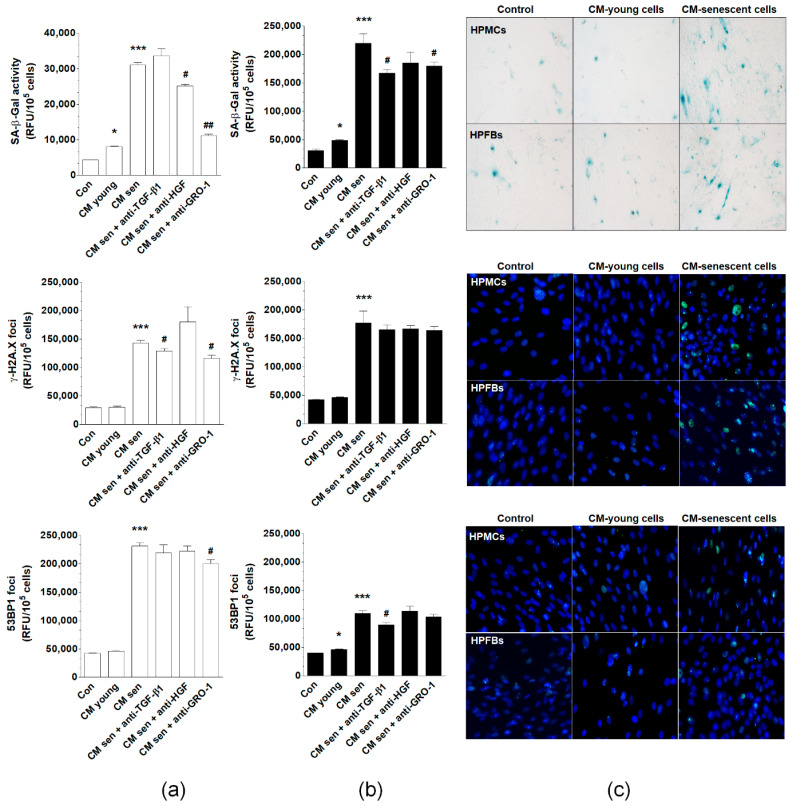
Effect of normal peritoneal mesothelial cells (HPMCs) and fibroblasts (HPFBs) on senescence induction in pEOCs. (**a**) Changes in SA-β-Gal, γ-H2A.X, and 53BP1 levels in pEOCs upon exposure to conditioned medium (CM) from young and senescent (**a**) HPMCs and (**b**) HPFBs. (**c**) Representative staining of SA-β-Gal, γ-H2A.X, and 53BP1 in pEOCs subjected to CM generated by HPMCs and HPFBs. Results are based on six independent experiments using pEOCs obtained from different patients. Results are expressed as mean ± SEM. * *p* < 0.05; *** *p* < 0.001 vs. Con.; ^#^ *p* < 0.05; ^##^ *p* < 0.01. vs. CM from senescent cells. RFU—relative fluorescence units.
